# Household Transmission of Pandemic (H1N1) 2009, San Antonio, Texas, USA, April–May 2009

**DOI:** 10.3201/eid1604.091658

**Published:** 2010-04

**Authors:** Oliver W. Morgan, Sharyn Parks, Trudi Shim, Patricia A. Blevins, Pauline M. Lucas, Roger Sanchez, Nancy Walea, Fleetwood Loustalot, Mark R. Duffy, Matthew J. Shim, Sandra Guerra, Fernando Guerra, Gwen Mills, Jennifer Verani, Bryan Alsip, Stephen Lindstrom, Bo Shu, Shannon Emery, Adam L. Cohen, Manoj Menon, Alicia M. Fry, Fatimah Dawood, Vincent P. Fonseca, Sonja J. Olsen

**Affiliations:** Centers for Disease Control and Prevention, Atlanta, Georgia, USA (O.W. Morgan, S. Parks, F. Loustalot, J. Verani, S. Lindstrom, B. Shu, S. Emery, A.L. Cohen, M. Menon, A.M. Fry, F. Dawood, S.J. Olsen); Department of State Health Services, Austin, Texas, USA (S. Parks, T. Shim, N. Walea, S. Guerra, V.P. Fonseca); United States Air Force School of Aerospace Medicine, Brooks City-Base, Texas, USA (P.M. Lucas, M.R. Duffy, M.J. Shim); San Antonio Metropolitan Health District, San Antonio, Texas, USA (P.A. Blevins, R. Sanchez, F. Guerra, B. Alsip); Comal County Health Department, New Braunfels, Texas, USA (G. Mills)

**Keywords:** Human influenza, viruses, pandemic (H1N1) 2009, household transmission, Texas, research

## Abstract

To assess household transmission of pandemic (H1N1) 2009 in San Antonio, Texas, USA, during April 15–May 8, 2009, we investigated 77 households. The index case-patient was defined as the household member with the earliest onset date of symptoms of acute respiratory infection (ARI), influenza-like illness (ILI), or laboratory-confirmed pandemic (H1N1) 2009. Median interval between illness onset in index and secondary case-patients was 4 days (range 1–9 days); the index case-patient was likely to be <18 years of age (p = 0.034). The secondary attack rate was 4% for pandemic (H1N1) 2009, 9% for ILI, and 13% for ARI. The secondary attack rate was highest for children <5 years of age (8%–19%) and lowest for adults >50 years of age (4%–12%). Early in the outbreak, household transmission primarily occurred from children to other household members and was lower than the transmission rate for seasonal influenza.

## Abstract 2

Transmission rates were lower than those for seasonal influenza.

On April 15 and 17, 2009, the first 2 cases of pandemic (H1N1) 2009 in the United States were identified among children in California; within 10 weeks, the strain was identified in 99 countries or territories (*1*). Texas was the second US state to confirm human transmission of pandemic (H1N1) 2009. On April 24, 2009, the Texas Department of State Health Services reported 2 patients with laboratory-confirmed pandemic (H1N1) 2009 infection in Guadalupe County. The strain was similar to that isolated previously from patients in Mexico and California (*2*). On June 11, 2009, the World Health Organization raised the pandemic alert to phase 6, indicating that a global pandemic was under way (*3*).

Characterizing transmission dynamics in various settings, such as households, schools, and the community, is critical to the development of appropriate guidance and public health interventions. Household contacts of persons with seasonal influenza are at increased risk for infection (*4**–**7*), but the household transmission characteristics of pandemic (H1N1) 2009 have yet to be fully characterized. This study reports household secondary attack rates and serial time intervals between illness onset in the index case-patient to illness onset in a household contact. We investigated persons with laboratory-confirmed pandemic (H1N1) 2009 and their household contacts in 1 health service region of Texas.

## Methods

### Population

The Texas Department of State Health Services consists of 11 health service regions. We conducted our investigation in Health Service Region 8, which includes 28 counties in south-central Texas, bordered on the south by Mexico (Figure). Approximately 2.4 million persons live in the region; 1.5 million live in the city of San Antonio (*8*). The Texas Department of State Health Services provides public health services in counties without local health departments. Within Health Service Region 8, public health services for Bexar County are provided by the San Antonio Metropolitan Health District and for Comal County by the Comal County Health Department.

### Case Definitions

We defined a laboratory-confirmed case-patient as a resident of Health Service Region 8 who had a positive respiratory specimen showing nucleic acid sequences unique to pandemic (H1N1) 2009; a real-time reverse transcription–PCR (rRT-PCR) assay was used to detect the virus (*9*). For persons with no laboratory test performed, we assessed whether they had influenza-like illness (ILI), defined as fever (measured or unmeasured) with either cough or sore throat; or acute respiratory infection (ARI), defined as >2 of the following signs or symptoms: fever, cough, sore throat, and rhinorrhea. The index case-patient was defined as the household member with the earliest symptom onset date of ARI, ILI, or laboratory-confirmed pandemic (H1N1) 2009. A secondary case-patient was defined as a household member with ARI, ILI, or laboratory-confirmed pandemic (H1N1) 2009 and symptom onset 1–9 days after symptom onset in the index case-patient. We chose the maximum interval of 9 days because shedding of seasonal influenza virus uncommonly lasts >8 days (*10*) and the median incubation period for seasonal influenza is ≈1.4 days (*11*). Household members were defined as persons who lived at the same address as a case-patient who had laboratory-confirmed pandemic (H1N1) 2009 infection.

### Case Finding

During April 10–May 8, 2009, we identified laboratory-confirmed cases of pandemic (H1N1) 2009 by reviewing 1,167 laboratory records of influenza specimens submitted by healthcare providers for rRT-PCR testing by the regional public health laboratory in San Antonio. We also reviewed 1,251 laboratory records of all specimens submitted by military medical treatment facilities in San Antonio. These specimens were tested for influenza by rRT-PCR at the Epidemiology Laboratory Service of the Department of Defense Global Influenza Surveillance Program at the United States Air Force School of Aerospace Medicine in San Antonio. In addition, we conducted telephone interviews with 540 (67%) of 802 high school students who were reported as absent by their school administrators during April 9–28 in the Texas counties where the first 2 identified case-patients attended school. Respiratory samples were collected from students who reported an acute respiratory illness at the time of interview. Additional case-patients were identified by collecting respiratory samples from nonhousehold contacts of laboratory-confirmed case-patients (i.e., those who had been within 6 feet of someone with ARI for at least 1 hour during the period 1 day before through 7 days after onset of illness in the contact).

### Household Investigations

We interviewed case-patients with laboratory-confirmed infection and all household members about the occurrence of illness, receipt of influenza vaccination in the previous 12 months, and medical history. We asked all persons about their use of antiviral medication and reviewed health department pharmacy records where appropriate to ascertain the type, dosage, and timing of antiviral medication and to define whether antiviral medications were prescribed for treatment or prophylaxis. Respiratory samples were collected from household contacts who had an acute respiratory illness at the time of interview; respiratory samples were collected from all members of 9 households identified early in the investigation, regardless of respiratory symptoms.

### Sample Collection and Laboratory Testing

Nasal wash samples were collected from military servicemen and their household family members, and nasopharyngeal swabs were collected from all others. Nasal wash samples were sent to the Epidemiology Laboratory Service at the United States Air Force School of Aerospace Medicine; nasopharyngeal swabs were sent to the regional public health laboratory in San Antonio. We used rRT-PCR to test all respiratory samples for seasonal influenza (A/H1 and A/H3 influenza viruses). Specimens positive for influenza A but negative for seasonal influenza by rRT-PCR were sent to the Centers for Disease Control and Prevention (CDC) for confirmatory testing for pandemic (H1N1) 2009 (*12*).

### Statistical Analysis

We calculated the serial interval as the number of days from the onset date of illness in the index case-patient to onset date of illness in the secondary case-patient. Secondary household attack rates were calculated by dividing the number of secondary case-patients (excluding the index case-patient) by the total number of household members (excluding the index case-patient). Secondary case-patients for ILI and ARI attack rates also included laboratory-confirmed case-patients. We compared characteristics between groups by using the χ^2^ test or Fisher exact test for categorical data and the Wilcoxon signed-rank test for continuous variables (*13*).

### Ethics

The collection of information about cases of pandemic (H1N1) 2009 was part of the emergency public health practice response and was not deemed to be research in accordance with the federal human subjects protection regulations (45 Code of Federal Regulations 46.101c and 46.102d) and CDC’s Guidelines for Defining Public Health Research and Public Health Non-Research. All protocols pertaining to the pandemic were reviewed for protection concerns and the necessity of Institutional Review Board review by the CDC’s National Center for Immunization and Respiratory Diseases (NCIRD) Human Subjects Contact and the NCIRD Associate Director of Science.

## Results

We identified 110 persons with laboratory-confirmed pandemic (H1N1) 2009 infection. We were unable to contact 23 (21%) of these persons, and 3 (3%) did not agree to provide further information. Of 84 persons with laboratory-confirmed pandemic (H1N1) 2009 infection who provided information, 77 (92%) lived with >1 persons. These 77 households comprised 349 persons; the median household size was 4 persons (range 2–9 persons), including the index case-patient. Seventy five percent of household interviews were conducted >8 days (range 0–24 days) after the onset of infection in the index case-patient.

From household interviews, we identified an additional 47 persons who reported respiratory symptoms or had laboratory evidence of pandemic (H1N1) 2009 infection: 13 persons with laboratory-confirmed pandemic (H1N1) 2009 infection, 24 persons with ILI, and 10 persons whose illness met the case definition for ARI only. We did not classify 15 of these persons as secondary case-patients: 8 persons had the same date of symptom onset as the index case-patient; we could not establish the date of symptom onset for 3 persons; and 4 persons reported illness onset 10–15 days after the index case-patient. In 1 household where 2 persons had ILI, 1 had a nasopharyngeal swab that was positive for pandemic (H1N1) 2009; the other was positive for influenza A, but the subtype could not be determined, possibly because of the quality of the sample or because 9 days had elapsed between illness and sample collection, thus decreasing viral load. We considered this person to have laboratory-confirmed pandemic (H1N1) 2009 on the basis of an epidemiologic link to another laboratory-confirmed case. In 2 households where secondary case-pateints were identified, nasal swab samples were obtained from members of all 7 households; 1 person, 14 years of age, who did not report any respiratory symptoms, was positive for pandemic (H1N1) 2009 infection.

Among the 97 symptomatic laboratory-confirmed case-patients (84 identified through case finding and 13 through household investigation), illness onset dates ranged from April 11 through May 8, 2009. Eleven (11%) were <4 years of age, 61 (63%) 5–18 years of age, 22 (23%) 19–49 years of age, and 3 (3%) >50 years of age. Forty-six (47%) were male. The most common signs and symptoms were fever (93%), cough (91%), rhinnorhea (70%), headache (67%), and sore throat (58%). Vomiting was reported by 26% and diarrhea by 25%. Ninety-two percent of laboratory-confirmed case-patients met the definition for ARI, and 85% met the definition for ILI. One laboratory-confirmed case-patient was hospitalized: a child who was admitted to the hospital with pneumonia in early April. No deaths occurred. Compared with household contacts who did not have laboratory-confirmed pandemic (H1N1) 2009 or did not report respiratory illness, laboratory-confirmed case-patients (index and secondary) were significantly younger (median age 17 vs. 24 years; p<0.001).

**Figure A1:**
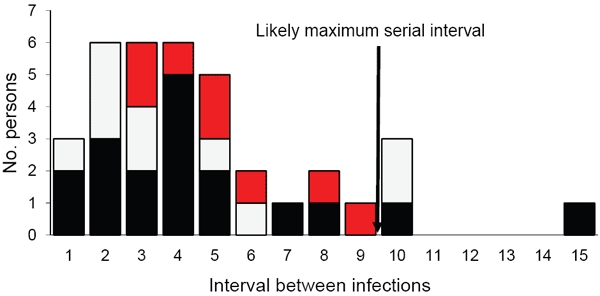
Interval between onset of illness for index and secondary case-patients (N = 32), Region 8, Texas, April–May 2009. Bars indicate number of case-patients who had acute respiratory infection (red bar sections), influenza-like illness (white bar sections), or laboratory-confirmed pandemic (H1N1) 2009 (black bar sections). Influenza-like illness, fever (measured or subjective), and cough or sore throat; acute respiratory infection, fever or cough or sore throat or rhinitis.

Secondary case-patients were found in 24 (31%) of 77 households; 5 had 2 secondary case-patients, and 1 had 3 case-patients (Table 1). Secondary infections appeared most likely to be transmitted between children (12/32, 38%) or children to adults (10/32, 31%) than from adults to children (6/32, 19%) or adults to adults (4/32, 13%) (p = 0.034). The median serial interval for ARI, ILI, and laboratory-confirmed pandemic (H1N1) 2009 combined was 4 days (range 1–9 days) (Table 1; Figure A1). Antiviral treatment was given to the index case-patient of 23 (72%) of 32 secondary case-patients; in these households, the serial interval was 3 days, compared with 5 days when the index case-patient was not given treatment (p = 0.17). Inclusion of 5 household contacts with illness that occurred 10–15 days after symptom onset of the index case-patient did not alter the median serial interval estimate. The median serial interval also remained unchanged when only members of households interviewed >9 days after the onset of symptoms in the household index case-patient were included. Limiting the estimate of median serial interval to include only persons with ILI or laboratory-confirmed case-patients reduced the median serial interval to 3 days (range 1–8 days).

**Table 1:** Index and secondary household case-patients with ARI, ILI, or laboratory-confirmed pandemic (H1N1) 2009, Region 8, Texas, April–May, 2009*

Household	Index case-patients				Secondary case-patients			Serial interval, d†
	Date of onset	Age, y	Case definition		Date of onset	Age, y	Case definition	
A	Apr 18	14‡	A, no subtype		Apr 25	21	Pandemic (H1N1) 2009	7
B	Apr 19	5	Pandemic (H1N1) 2009		Apr 21	9	Pandemic (H1N1) 2009	2
C	Apr 22	18	Pandemic (H1N1) 2009		Apr 25	<1	Pandemic (H1N1) 2009	3
D	Apr 26	1	Pandemic (H1N1) 2009		May 4	27	Pandemic (H1N1) 2009	2
E	Apr 26	16	Pandemic (H1N1) 2009		Apr 27	51	Pandemic (H1N1) 2009	1
					Apr 27	8	ILI	1
F	Apr 27	<1	Pandemic (H1N1) 2009		Apr 29	22	Pandemic (H1N1) 2009	2
					May 6	47	ARI	9
G	Apr 27	16	Pandemic (H1N1) 2009		May 1	16	Pandemic (H1N1) 2009	4
					May 1	14	Pandemic (H1N1) 2009	4
H	Apr 29	6	Pandemic (H1N1) 2009		Apr 3	<1	Pandemic (H1N1) 2009	1
I	May 3	33	Pandemic (H1N1) 2009		May 7	15	Pandemic (H1N1) 2009	4
					May 8	14	Pandemic (H1N1) 2009	5
Subtotal no. case-patients			9				13	3 (1–9)
J	Apr 20	17	Pandemic (H1N1) 2009		Apr 26	14	ARI	6
K	Apr 24	71	Pandemic (H1N1) 2009		Apr 27	65	ILI	3
L	Apr 25	16	Pandemic (H1N1) 2009		Apr 27	16	ILI	2
M	Apr 25	12	Pandemic (H1N1) 2009		Apr 28	30	ARI	3
					Apr 28	33	ARI	3
					Apr 30	6	ARI	5
N	Apr 26	30	Pandemic (H1N1) 2009		May 1	28	ARI	5
O	Apr 27	33	Pandemic (H1N1) 2009		May 5	53	ARI	8
P	Apr 28	25	Pandemic (H1N1) 2009		May 4	14	ILI	6
Q	Apr 29	1	Pandemic (H1N1) 2009		May 1	21	ILI	2
					May 2	2	ILI	3
R	Apr 29	8	Pandemic (H1N1) 2009		May 3	44	ARI	4
S	May 1	6	Pandemic (H1N1) 2009		May 3	45	ILI	2
Subtotal no. case-patients			10				13	3 (1–8)
T	Apr 17	11	ILI		Apr 21	18	Pandemic (H1N1) 2009	4
U	Apr 18	48	ILI		Apr 26	10	Pandemic (H1N1) 2009	8
V	Apr 23	53	ILI		Apr 26	42	Pandemic (H1N1) 2009	3
W	Apr 24	5	ILI		Apr 29	<1	Pandemic (H1N1) 2009	5
X	Apr 28	26	ILI		May 2	7	Pandemic (H1N1) 2009	4
					May 3	4	ILI	5
Subtotal no. case-patients			5				6	4.5 (3–8)
Total no. case-patients			24				32	4 (1–9)

*ARI, acute respiratory infection; ILI, influenza-like illness (fever measured or subjective and cough or sore throat). †Median (range) number of days between symptom onset of the index and secondary case-patients. ‡The influenza virus from this person could not be subtyped, possibly because of the quality of the sample or the length of time from symptom onset to sample collection. We considered this case-patient to have been infected with pandemic (H1N1) 2009.

The secondary household attack rate was 13% for ARI, 9% for ILI, and 4% for laboratory-confirmed pandemic (H1N1) 2009 (Table 2). Secondary attack rates were highest in children <5 years of age and were higher in children 5–18 years of age than in adults 19–49 and >50 years of age (Table 2). By household size, secondary attack rates for ARI, ILI, and laboratory-confirmed pandemic (H1N1) 2009 were highest in households with 2–3 persons (ARI 23%, ILI 23%, laboratory-confirmed pandemic [H1N1] 2009 6%) and were lowest in households with 7–9 persons (ARI 9%, ILI 9%, laboratory-confirmed pandemic [H1N1] 2009 2%) (Table A1). The secondary household attack rate did not differ by receipt of seasonal influenza vaccination in the previous 12 months (Table A2).

**Table 2:** Household secondary attack rates for ARI, ILI, and laboratory-confirmed pandemic (H1N1) 2009, by age group, Region 8, Texas, April–May 2009*

Illness type by age group, y	No. index case-patients	Household contacts			Household members not included	Secondary attack rate (A/A + B), %
		Secondary case-patients, A	Not ill, B	Total household contacts, A + B		
ARI						
<5	7	5	23	28	1	18
5–18	50	13	83	96	3	14
19–49	17	11	96	107	3	10
>50	3	3	22	25	1	12
All ages	77	32	224	256	8	13
ILI						
<5	6	5	23	28	2	18
5–18	50	11	86	97	2	11
19–49	18	6	102	108	1	6
>50	3	2	23	25	1	8
All ages	77	24	234	258	6	9
Laboratory-confirmed pandemic (H1N1) 2009						
<5	8	2	26	28	0	7
5–18	51	5	92	97	1	5
19–49	16	3	108	111	0	3
>50	2	1	26	27	0	4
All ages	77	11	252	263	1	4

*ARI, acute respiratory infection; ILI, influenza-like illness (fever measured or subjective and cough or sore throat). Ill household members were not included in the calculation of the secondary attack rate if they had the same symptom onset as the index case or if symptom onset was not known.

Treatment with antiviral medication was given to 77% of index case-patients (57/74 of persons with ARI, ILI, and laboratory-confirmed pandemic [H1N1] 2009 combined for whom data were available) and 72% of secondary cases (23/32 of ARI, ILI, and laboratory-confirmed pandemic [H1N1] 2009 combined); 90% took oseltamivir; 7% took zanamivir; and 3% took an unknown type of antiviral medication. Neither the age of the index case-patient, household size, nor diagnosis of the index patient (with ARI, ILI, or laboratory-confirmed pandemic [H1N1] 2009) were predictive of treatment with antiviral medication. The secondary household attack rates for ARI, ILI, and laboratory-confirmed pandemic (H1N1) 2009 combined in households where the index case-patient was given antiviral treatment was 12% compared with 16% in other households (p = 0.64) (Table 3). Antiviral prophylaxis was given to 39% of household contacts (92/235 with data available) (Table 3), and the secondary attack rate of ARI, ILI, and laboratory-confirmed pandemic (H1N1) 2009 combined was 14% (12/83) in households where the index patient took treatment, compared with 66% (6/9) (p = 0.003) in households where the index patient did not take treatment (Table 3).

**Table 3:** Household secondary attack rates for ARI, ILI, and laboratory-confirmed pandemic (H1N1) 2009, by antiviral medication treatment and prophylaxis, Region 8, Texas, April–May 2009*

Type of contact	No. contacts	Index case-patient received antiviral treatment (attack rate, %)		
		Yes	No	p value†
All contacts of index case-patients	235	22/185 (12)	8/50 (16)	0.64
Contacts who took antiviral prophylaxis	92	12/83 (14)	6/9 (67)	0.003

*ARI, acute respiratory infection; ILI, influenza-like illness (fever measured or subjective and cough or sore throat). †Fisher exact test comparing the secondary attack rate for any treatment to no antiviral treatment. Data about antiviral medication were missing for 2 index case-patients and 15 contacts.

## Discussion

During an outbreak of pandemic (H1N1) 2009 in the San Antonio, Texas, area, we identified 97 persons with laboratory-confirmed infection in 77 households. The epidemiologic and clinical features were similar to summary reports from the United States (*14**,**15*) and other countries (*15**,**16*). Nearly one third of households had secondary case-patients who also had respiratory illness, with a median of 4 days between onset of illness in the index case-patient and household members, a finding similar to that for seasonal influenza (*17*).

The secondary attack rate was 4% for laboratory-confirmed pandemic (H1N1) 2009, 9% for ILI, and 13% for ARI. In general, these rates are lower than for seasonal influenza and lower than anticipated for a pandemic strain, although rates vary from 13% to 30%, depending on influenza subtype and year and pandemic period (*4**,**5**,**18**–**21*). The highest proportion of laboratory-confirmed pandemic (H1N1) 2009 and secondary attack rates occurred in children, a finding consistent with the epidemiology of seasonal and pandemic influenza, where we know children experience higher rates of illness (*4**,**5**,**7*) and higher secondary attack rates (*19*). Adults may have some cross-protection against pandemic (H1N1) 2009 from antibodies developed during infections with seasonal influenza A virus (H1N1) (*22**–**24*).

Four randomized controlled trials of zanamivir and oseltamivir for seasonal influenza have shown that these antiviral medications reduce but do not eliminate viral shedding and are effective in preventing disease among household contacts, especially if taken within 48 hours of illness onset in the index case-patient (*19**,**20**,**25**,**26*). We found that secondary attack rates for all households were lower when the index case-patient received treatment, although this difference was not significant. The role of prophylaxis in the absence of treatment of the index case-patient was difficult to determine; our investigation included only a small number of such persons. Nevertheless, because most index and secondary case-patients received antiviral treatment, household secondary attack rates may have been reduced.

Our investigation has several limitations. Because early case finding was most intensive among high school children associated with school outbreaks, our cohort may have been biased in favor of households where the index case-patients were children; however, this would not explain a lower secondary attack rate among adult household contacts. We did not assess the role of mild or asymptomatic pandemic (H1N1) 2009 infection because we collected respiratory samples only; serologic assays to detect influenza antibodies are the most sensitive method for detecting asymptomatic infection, but virus assays for pandemic (H1N1) 2009 were not available at the time of the investigation. Volunteer challenge studies with seasonal influenza viruses have found that up to 30% of infected persons may be asymptomatic and could be identified through serologic testing (*10*). Because 25% of household interviews were conducted <8 days after onset of illness of the index case-patient, we may have underestimated the secondary attack rate if these households had secondary case-patients with long serial intervals. However, when we restricted our analysis to persons interviewed >8 days after onset of symptoms in the index case-patient, we found no difference in the median serial interval or distribution of attack rates by age. Conversely, household members interviewed >8 days after onset of illness in the index case-patient may have had incomplete recall of acute respiratory infections. Finally, some of the secondary illnesses may have been acquired in the community, leading to overestimate of household secondary attack rates.

We found that pandemic (H1N1) 2009 disproportionately affected children, who in turn posed a risk for secondary household transmission, especially to their caregivers and siblings. The Advisory Committee on Immunization Practices (2009) recommends that children 6–18 years of age and caregivers of infants be included as initial target groups for the new pandemic (H1N1) 2009 vaccine (*27*), which may reduce household transmission. As pandemic (H1N1) 2009 continues to spread internationally, ongoing investigations are needed to shed further light on transmission dynamics, to monitor epidemiologic changes over time, and to assess the effectiveness of public health interventions.

## Figures and Tables

**Figure:**
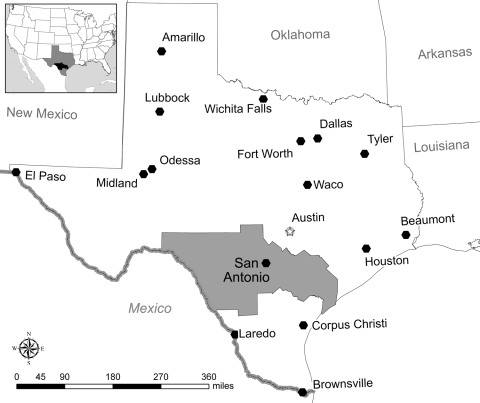
Texas Department of State Health Services Health Service Region 8 (gray shading), Texas, USA.

## Appendix

**Table A1:** Household secondary attack rates for ARI, ILI, and laboratory-confirmed pandemic (H1N1) 2009, by household size, Region 8, Texas, April–May 2009*

Household size, no. persons	Index case-patients	Household contacts			Ill household members not included	Secondary attack rate, A/A + B, %
		Secondary case-patients, A	Not ill, B	Total household contacts, A + B		
ARI						
2–3	21	7	24	31	1	23
4	23	11	58	69	0	16
5–6	26	10	101	111	4	9
7–9	7	4	41	45	3	9
All households	77	32	224	256	8	13
ILI						
2–3	21	7	24	31	1	23
4	23	8	61	69	0	12
5–6	26	5	107	112	3	4
7–9	7	4	42	46	2	9
All households	77	24	234	258	6	9
Laboratory-confirmed pandemic (H1N1) 2009						
2–3	21	2	30	32	0	6
4	23	4	65	69	0	6
5–6	26	4	110	114	1	4
7–9	7	1	47	48	0	2
All households	77	11	252	263	1	4

*ARI, acute respiratory infection; ILI, influenza-like illness (fever measured or subjective and cough or sore throat). Ill household members were not included in the calculation of the secondary attack rate if they had the same symptom onset as the index case or if symptom onset was not known.

**Table A2:** Household secondary attack rates for ARI, ILI, and laboratory-confirmed pandemic (H1N1) 2009, by seasonal influenza vaccination status, Region 8, Texas, April–May 2009*

Received seasonal influenza vaccine in past 12 mo	Index case-patients	Household contacts			Ill household members not included	Secondary attack rate, A/A + B, %
		Secondary case-patients, A	Not ill, B	Total household contacts, A + B		
ARI						
Vaccinated	23	8	49	57	2	14
Not vaccinated	52	21	167	188	6	11
Vaccine status unknown	2	3	8	11	0	–
All households	77	32	224	256	8	13
ILI						
Vaccinated	22	6	51	57	3	11
Not vaccinated	53	15	175	190	3	8
Vaccine status unknown	2	3	8	11	0	–
All households	77	24	234	258	6	9
Laboratory-confirmed pandemic (H1N1) 2009						
Vaccinated	22	1	56	57	1	2
Not vaccinated	53	8	188	196	0	4
Vaccine status unknown	2	2	8	10	0	–
All households	77	11	252	263	1	4

*ARI, acute respiratory infection; ILI, influenza-like illness (fever measured or subjective and cough or sore throat). Ill household members were not included in the calculation of the secondary attack rate if they had the same symptom onset as the index case or if symptom onset was not known.
